# Potentials and Challenges of Genomics for Breeding Cannabis Cultivars

**DOI:** 10.3389/fpls.2020.573299

**Published:** 2020-09-25

**Authors:** Gianni Barcaccia, Fabio Palumbo, Francesco Scariolo, Alessandro Vannozzi, Marcello Borin, Stefano Bona

**Affiliations:** DAFNAE–Department of Agronomy, Food, Natural Resources, Animals and Environment, University of Padova, Campus of Agripolis, Legnaro, Italy

**Keywords:** *Cannabis sativa*, breeding methods, F_1_ hybrids, simple sequence repeat markers, DNA barcodes, MADS box genes

## Abstract

*Cannabis* (*Cannabis sativa* L.) is an influential yet controversial agricultural plant with a very long and prominent history of recreational, medicinal, and industrial usages. Given the importance of this species, we deepened some of the main challenges—along with potential solutions—behind the breeding of new cannabis cultivars. One of the main issues that should be fixed before starting new breeding programs is the uncertain taxonomic classification of the two main taxa (e.g., *indica* and *sativ*a) of the *Cannabis* genus. We tried therefore to examine this topic from a molecular perspective through the use of DNA barcoding. Our findings seem to support a unique species system (*C. sativa*) based on two subspecies: *C. sativa* subsp. *sativa* and *C. sativa* subsp. *indica*. The second key issue in a breeding program is related to the dioecy behavior of this species and to the comprehension of those molecular mechanisms underlying flower development, the main cannabis product. Given the role of MADS box genes in flower identity, we analyzed and reorganized all the genomic and transcriptomic data available for homeotic genes, trying to decipher the applicability of the ABCDE model in *Cannabis*. Finally, reviewing the limits of the conventional breeding methods traditionally applied for developing new varieties, we proposed a new breeding scheme for the constitution of F_1_ hybrids, without ignoring the indisputable contribution offered by genomics. In this sense, in parallel, we resumed the main advances in the genomic field of this species and, ascertained the lack of a robust set of SNP markers, provided a discriminant and polymorphic panel of SSR markers as a valuable tool for future marker assisted breeding programs.

## 1. General Introduction to *Cannabis* spp.: Taxonomy and History of Cultivated Varieties

*Cannabis sativa* L. is an agricultural plant species that today enjoys great interest because of its multiple uses in the recreational, medicinal, and industrial areas (Kovalchuk et al., 2020). This plant can be cultivated for the production of fibers (used to make different textiles), seeds (rich in unsaturated fatty acids for edible oils), and drugs from its female inflorescences that contain cannabinoids (compounds with psychotropic or psychopharmaceutical effects). Among these latter, the principal psychoactive constituent of cannabis is THC (tetrahydrocannabinol), and the concentration of this metabolite is at the basis of the distinction between hemp and drug (marijuana) types, with hemp considered low in concentration, 0.3% or less THC content (non-psychoactive), and marijuana, on the other hand, containing up to 30% THC by dry weight. In the present review, we will mainly focus on drug type cannabis.

The genus *Cannabis* belongs to the family of Cannabaceae (order Rosales). Its botanical classification had a very troubled genesis since the times of Linnaeus considering it was not clear whether the genus was mono- or polytypic (Schultes, 1970; Small and Cronquist, 1976; Schultes and Hofmann, 1980). In 1597, John Gerarde (Gerarde, 1597) first defined the plant species as dioecious, but the question remained open because monoecious plants can occur and hermaphroditism is also possible with plants that show reproductive organs within the same flower (Small and Cronquist, 1976; Clarke, 1981; Ming et al., 2011). All these biological variants are known to be very frequent in fiber varieties (Small and Cronquist, 1976). Plants also manifest sexual dimorphism, with male individuals being often characterized by a shorter crop cycle and a taller stature than female ones. Lamarck originally recognized two interfertile species *C. sativa* (from Persia) and *C. indica* (from India) (Lamarck, 1785). Based on this old taxonomy, many varieties available on the market are still classified as *C. sativa* × *C. indica* hybrids. As a matter of fact, the reproductive system of cannabis plants is characterized by allogamy and anemophily, and therefore open pollination is necessarily responsible for a certain degree of hybridization between improved and wild populations. This is why, according to Schultes, landraces of cannabis should no longer exist since several decades (Schultes, 1970). Later on, Small and Cronquist (Small and Cronquist, 1976) proposed a unique species system that is still widely accepted and that is based on two subspecies of *C. sativa*: *C. sativa* subsp. *sativa* and *C. sativa* subsp. *indica*. Although several authors, supporting the one-species system for cannabis, recommend to classify its varieties based on the cannabinoids and terpenoids profile (Hazekamp et al., 2016; Piomelli and Russo, 2016), a molecular system based on DNA barcoding could represent a cost- and time-effective technique of great help in clarifying some of the taxonomic issues related to the genus *Cannabis*. DNA barcoding could also play a crucial role in the identification and characterization of those uncertified cannabis strains, which are mainly derived from black market. Section 2 reviews the DNA barcoding data available for this genus and explores the potential use of this technique for taxonomic identity surveys.

**Figure 1:**
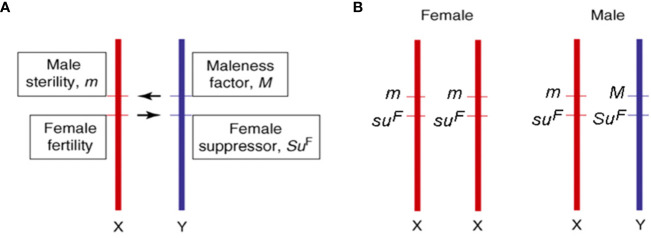
Information on sex determinants **(A)** and sex chromosomes **(B)** in cannabis [adapted from (Bergero and Charlesworth, 2008; Divashuk et al., 2014)].

According to Charlesworth et al. (2005), the dioecious species evolved from a common monoecious ancestor shared by *Cannabis* and *Humulus* (Kovalchuk et al., 2020) both characterized by having sex chromosomes (Renner, 2014). In particular, *C. sativa* possesses nine pairs of autosomes and a pair of X and Y sex chromosomes. The male sex is heterogametic (XY), while the female is homogametic (XX), and different authors reported distinct mechanisms involved in the determination of sex (Sakamoto et al., 1998; Faux et al., 2016). This uncertainty could derive from the fact that environmental conditions, and in particular abiotic stress factors, can influence the expression and the determination of sex (Vergara et al., 2016a). Although the structure of sex chromosomes is poorly understood in *Cannabis* spp., since it is not detectable with standard microscopic techniques (Sakamoto et al., 1998; Peil et al., 2003), the Y chromosome was shown to have larger dimensions than the X chromosome (Sakamoto et al., 1998; van Bakel et al., 2011). More recently, both male and female karyotypes of *C. sativa* L. were extensively characterized by DAPI banding procedures and FISH analyses using rDNA probes (Divashuk et al., 2014). Sex determination represents one of the main problems when breeding new cannabis varieties since it can only be assessed at the beginning of flowering, when male and female flowers are visible and distinguishable. The genetic control of dioecy seems to be determined by two specific genes at linked loci acting as sex determinants (Bergero and Charlesworth, 2008; Divashuk et al., 2014; Henry et al., 2018): Male plants would require a dominant suppressor of female organs (Su^F^) and a dominant activator of maleness (M), while female plants would share homozygosity for their recessive alleles at both loci (su^F^su^F^ mm), as illustrated in **Figure 1**. For breeding purposes, male and female plants can then be identified in the early stages of development through the use of Y-specific DNA markers (Mandolino et al., 1999; Törjék et al., 2002). Apart from that, the molecular mechanisms underlying dioecy are essentially unknown but, considering that this condition is fully reversible (e.g., through chemical products treatment), the hypothesis that those genic regions involved in both sexes development remain potentially functional throughout the entire life cycle cannot be excluded (Di Stilio et al., 2005; Khadka et al., 2019). Given the role of homeotic genes in flower whorls identity (including anthers, pistils, and ovary), the hypothesis for their involvement in sex determination (Pfent et al., 2005; Sather et al., 2010; LaRue et al., 2013) and the lack of any information on the ABCDE model in the *Cannabis* genus, we screened all cannabis genomic and transcriptomic data available for homeotic genes and summarized them in Section 3. Traditionally, hemp-type and drug-type varieties have been bred mainly through mass selection. This method has been effectively used for the selection of cannabis showing improved quality traits such as fiber, oil, and cannabinoid content (Hennink, 1994). Nevertheless, one of the main problems associated with the first attempts of cannabis genetic improvement was, on the one hand, the need to avoid hemp genotypes with high THC contents, on the other hand, the availability of uniform medical genotypes, which was often linked to clandestine growers. More recently, cannabis cultivars were obtained from controlled mating using selected individuals from different landraces and cultivars. Usually, several selected individuals were used for open-pollination so that each of the female plants could be fertilized by each of the male plants (i.e., intercrosses). Synthetic varieties were also obtained by open-pollination using many female and male plants vegetatively propagated *via* cuttings (i.e., polycrosses).

**Figure 2:**
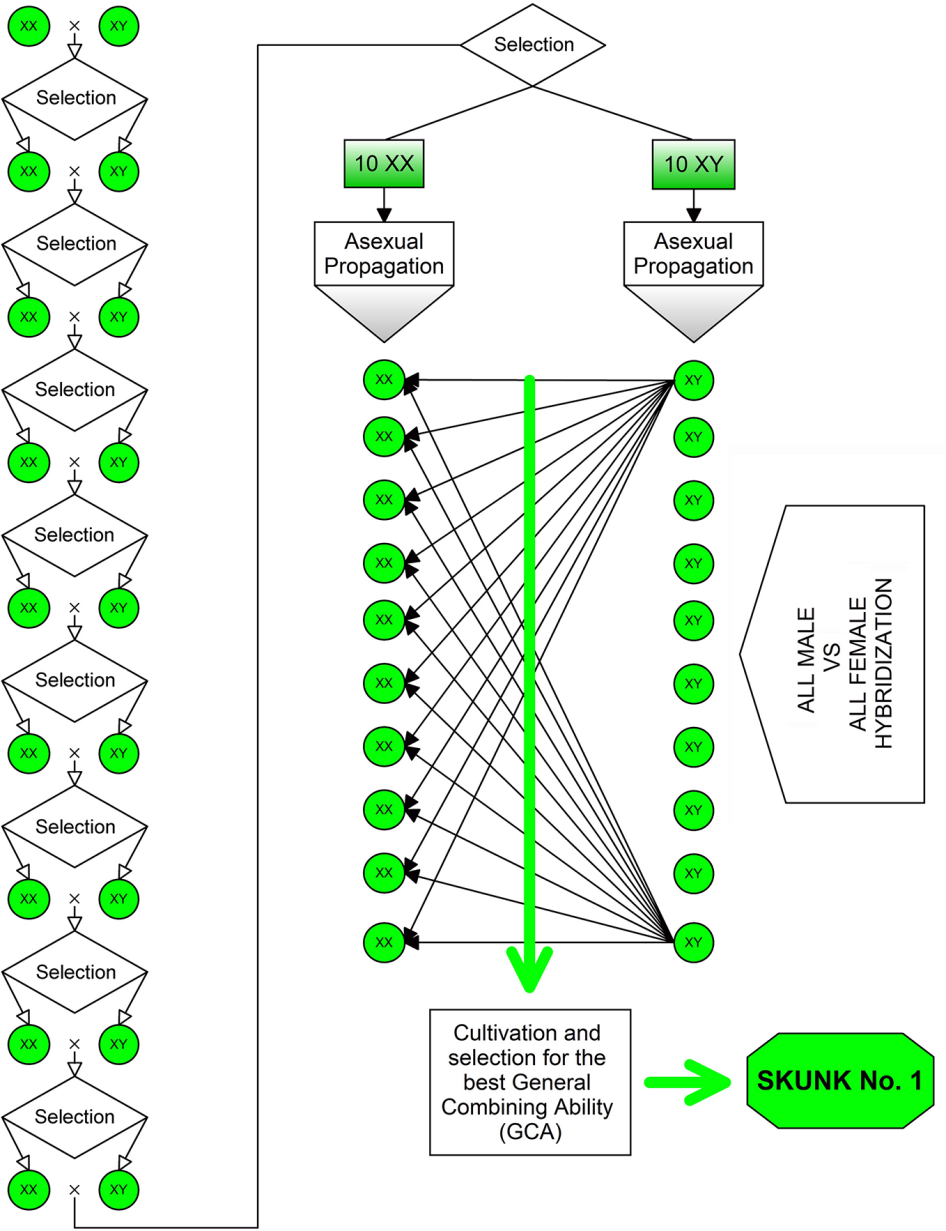
Method used for the development of the “Skunk No. 1”: the first NLD/BLD hybrid bred in the early 1970s. To obtain this variety, plants of the F_2_ progeny were chosen to carry out nine repeated inbreeding cycles aimed at increasing their homozygosity, then ten female and ten male plants were selected and vegetatively propagated to be used as parental lines in all possible pairwise cross-combinations.

Heterosis (or hybrid vigor) has been a driving factor for breeding programs aimed at the development of both modern fiber- and drug-type cultivars. The heterotic effect is usually manifested by highly heterozygous plants produced by crossing two different lineages and/or antagonist genotypes (i.e., using parental lines that show high homozygosity for antagonist gene forms across most of the loci). The first NLD/BLD (Narrow Leaflet Drug/Broad Leaflet Drug) hybrid was “Skunk No. 1” produced in the early 1970s (**Figure 2**). To obtain this variety, plants of the F_2_ progeny were chosen to carry out nine repeated inbreeding cycles aimed at increasing their homozygosity, then ten female and ten male plants were selected and vegetatively propagated for use as parental lines in all possible pairwise cross-combinations. Such a breeding strategy is very effective for the development of highly heterozygous synthetic varieties, especially if supported by progeny tests to assess the general combining ability (GCA) of parental lines.

More frequently, selected F_1_ plants have been used to generate large segregating F_2_ populations from which favorable individuals could be eventually cloned *via* cuttings or used in half- or full-sibling matings. Cultivated varieties, or cultivars, were mainly produced by crossing a single male of one genetically distinct landrace with a single female of another landrace to create a hybrid, heterozygous and vigorous offspring. In the subsequent F_1_ generation, selected male or female progenies were bred by following one of these basic strategies: 1) Plants were inbred with one or more siblings to establish a relatively heterozygous or highly heterogeneous F_2_ population to be used in subsequent mass selection cycles to increase homozygosity and uniformity by intercrossing selected plants; 2) plants were backcrossed with a parental line (the seed parent or the pollen donor) to recover and fix specific traits before establishing mass selection; or 3) plants were outcrossed with an unrelated line (a plant from a third landrace) to integrate new traits and create new recombinants. Each of these breeding strategies was efficiently used to develop new cultivars using experimental hybrid materials that stemmed from crosses between distinct landraces. However, true F_1_ hybrid varieties were never bred in the past since agronomically super-pure inbred lines to be used yearly as parental lines were difficult to implement. Only recently some professional seed companies have produced and multiplied true F_1_ hybrid varieties by preserving vegetatively parental clones of the male and female lines. Nevertheless, if the parental clones are not fully homozygous and so genetically unstable, their hybrid progeny is frequently inconsistent phenotypically because of the genetic segregation of maternal and/or paternal traits. As a matter of fact, most seed companies invest in breeding programs aimed at selecting superior female plants, while male plants are deriving from the standard morphological analysis: an individual male is then used as a pollen donor in crosses performed with each of the female clones to produce commercial hybrid seed stocks. These seeds, which do not have the genetic constitution of F_1_ hybrids, are then widely distributed and grown to maturity so that female plants can be selected and multiplied by cuttings to achieve commercial sinsemilla production. In recent years, seeds of the so-called “all-female” cultivars have been largely set by promoting artificially selfing: this is possible by applying hormones to some branches of female plants to let them produce also male flowers with viable genetically female pollen. As a consequence, the offspring of female plants fertilized with female pollen of masculinized branches include only genetically female progeny. This is a very efficient strategy for commercial sinsemilla production as all seeds generate useful female plants with no need to remove male plants, so it provides the benefits of asexual propagation (i.e., fixation of the female genotype), but with the advantages of sexual reproduction (i.e., reproduction *via* seeds in place of cuttings). However, female seeds can give rise to unstable populations characterized by some degree of genetic diversity, in contrast to clonal populations produced from female cuttings. In fact, under sexual reproduction, segregation and recombination mechanisms are all possible unless the parental lines are highly homozygous inbred lines suitable for breeding true F_1_ hybrids. For this reason, Section 4 of this review offers new insights on next-generation methods for breeding new and true cannabis F_1_ hybrids.

Nevertheless, it cannot be overlooked that breeding methods conventionally used for the development of new varieties have been revolutionized since the advent of genomics applied to crop plant species. In fact, the examination of plant materials using molecular markers linked to single loci controlling specific traits of agronomic interest (i.e., marker-assisted selection, MAS) and the exploitation of multiple loci genotyping with molecular markers scattered throughout the genome (i.e., marker-assisted breeding, MAB) provide the opportunity to boost gain from selection (Tuberosa, 2012). For this reason, Section 5 provides an analytical review of the main achievements reached by genomics applied to plant resources of the genus *Cannabis*. Lastly, owing to the lack of a robust panel of SNP markers based on a standardized set of genes and considering the urgent need to develop a reference method for genotyping plant varieties with ease to detect markers, as well as reliable and transferable protocols, a discriminant panel of SSR markers was selected from polymorphic microsatellite regions of *Cannabis* spp. Recent signs of progress in the development of multiplex assays have been made in several crops (Palumbo et al., 2018; Patella et al., 2019a; Patella et al., 2019b), suggesting that these markers, especially when finely mapped and scattered throughout the genome, remain as relevant and cost-effective molecular tools at least for characterizing genetic resources and breeding new varieties. On the whole, this information is reported in Section 6.

## 2. Chloroplast DNA Barcodes and ITS Regions for *Cannabis* Species Authentication: What Is Available and Retrievable From Public Nucleotide Repositories

Currently, with the cannabis market showing increases in both demand and availability and cannabis seed companies arising wherever national law allows it, the necessity for a reliable molecular based-taxonomic system for this species is urgent. Many cannabis cultivars are obtained by crossing plants from what are commonly considered subspecies. In general, lines belonging to the two main subspecies of *C. sativa*, subsp. *sativa* and subsp. *indica* (Small and Cronquist, 1976), are used to produce new varieties suitable for different uses, such as fiber, oil, medical drug, and recreational applications. These subspecies differ in phenotype and chemotype, and the main characteristics according to which they are commonly distinguished are size, leaf shape, terpene accumulation, the quantity and chemistry of cannabinoids produced and earliness of flowering. A great amount of interest from breeders is focused on the determination of the subspecies “composition” of the parental lines used in crosses and that of the obtained offspring. It is important to consider the origin and phylogeny of a line or cultivar to better plan breeding strategies and guarantee a higher level of traceability. Whether for medical or recreational use, costumers are increasingly interested in tracing the origins of the products they use. Although much information about the phylogenetic taxonomy of this species is available, it is often controversial. In 2018, McPartland (2018) highlighted the different nomenclatures applied to this plant over time, from Linneus and Lemarck in the 18^th^ century to the most recent classification proposed by the Angiosperm Phylogeny Group in the 21^st^ century (The Angiosperm Phylogeny Group, 2003).

The common molecular approach for the taxonomic determination of a species or subspecies is to apply DNA barcoding to the extra-nuclear genome. In animal species, the cytochrome *c* oxidase I (*cox*I) mitochondrial gene has been set by the “Consortium of Barcode of Life” as a standard DNA barcode for determining the phylogenetic relationships between organisms, and Hebert (Hebert et al., 2003) proposed a threshold of a genetic difference in the *cox*I region equal to 2.7% for the discrimination of animal species. Since the *cox*I gene is not suitable for discriminating different taxa due to a low mutation rate in the plant mitochondrial genome, in 2007, Kress and Erickson (2007) demonstrated the suitability of the Ribulose-1,5-bisphosphate carboxylase/oxygenase large subunit (*rbc*L) gene and *trn*H-*psb*A noncoding spacer region as DNA barcodes for plant classification. Later, the maturase K (*mat*K) gene was included in the list of exploitable markers for DNA barcoding in land plants (Asahina et al., 2010; Dunning and Savolainen, 2010; de Vere et al., 2015). Moreover, as the classification efficacy of these barcodes has sometimes been demonstrated to not be sufficiently informative, the use of other regions, both plastidial and nuclear, such as *rpoC1* and *ycf5*, and ITS1 and ITS2, respectively, has been proposed for this purpose (Chen et al., 2010; Wang et al., 2014).

Much conflicting information regarding the taxonomic classification of *C. sativa* is available in the scientific literature (Lightfoot et al., 2016; McPartland, 2018), and the debate regarding its possible subdivision into different subspecies is still open. Because of this, we reviewed the DNA barcoding data (i.e., ITS1, ITS2, *mat*K and *rbc*L sequences) available for cannabis in the two main public repositories (BOLD and GenBank) (Ratnasingham and Hebert, 2013) through a keyword search and BLASTn analysis for “*Cannabis*” taxa (taxid: 3482).

A total of 112 sequences were collected, including 15 *mat*K (only *C. sativa*), 59 *rbc*L (only *C. sativa*), 12 ITS1 (10 C*. sativa* and 2 C*. sativa* subsp. *indica*) and 26 ITS2 (23 C*. sativa*, 2 C*. sativa* subsp. *indica* and 1 *Cannabis ruderalis*) sequences, which were aligned for each gene using the Geneious software Clustal Omega plug-in (Sievers et al., 2011) to investigate the percentage of pairwise identity within and between the taxa for which multiple sequences were available (i.e., ITS1 and ITS2) (**Table 1** and details in **Supplementary Table 1**).

**Table 1:** Sequences retrieved from BOLD/NCBI databases of the chloroplast genes *mat*K and *rbc*L and nuclear regions ITS1 and ITS2.

N. seqs cpDNA	Taxa	Pairwise identity (%)	Barcode gene	
15	*Cannabis sativa*	99.7%	*mat*K	
59	*Cannabis sativa*	99.6%	*rbcL*	
**N. seqs ITS1**	**Taxa**	*Cannabis sativa*	*Cannabis sativa* subsp. *indica*	*Cannabis ruderalis*
10	*Cannabis sativa*	99.9%		
2	*Cannabis sativa* subsp. *indica*	99.9%	100.0%	
0	*Cannabis ruderalis*	N/A	N/A	N/A
**N. seqs ITS2**	**Taxa**	*Cannabis sativa*	*Cannabis sativa* subsp. *indica*	*Cannabis ruderalis*
23	*Cannabis sativa*	99.8%		
2	*Cannabis sativa* subsp. *indica*	99.8%	99.5%	
1	*Cannabis ruderalis*	99.8%	99.7%	N/A

Pairwise identity percentages within and between taxa (where possible, in triangular matrixes) are also reported. N/A, number of sequences insufficient for the analysis.

Chloroplast genes were available only for the *C. sativa* taxa, and none were found for subsp. *indica* or *ruderalis*, making it impossible to compare them. Despite this, the calculated within-taxon (i.e., within *C. sativa)* percentages of identity were 99.7% and 99.6% for *mat*K and *rbc*L, respectively.

On the other hand, the nuclear regions showed levels of identity within the same taxa of 100% (*C. sativa* subsp. *indica*) and 99.9% (*C. sativa*) for ITS1, while they were equal to 99.8% (*C. sativa*) and 99.5% (*C. sativa* subsp. *indica*) for ITS2. Regarding the sequence identity between *C. sativa* subsp. *indica* and *C. sativa*, it resulted 99.9% for ITS1 and 99.8% for ITS2 (**Table 1**).

The only sequence available for *C. ruderalis* (ITS2) was used for a comparison between taxa, with values of 99.8% (*C. ruderalis* vs *C. sativa)* and 99.7% (*C. ruderalis* vs *C. sativa* subsp. *indica*; **Table 1**).

## 3. Genomics of Flower Organ Identity in Cannabis: A Comprehensive In Silico Survey of the ABCDE Genes Encoding MADS-Box Transcription Factors

**Figure 3:**
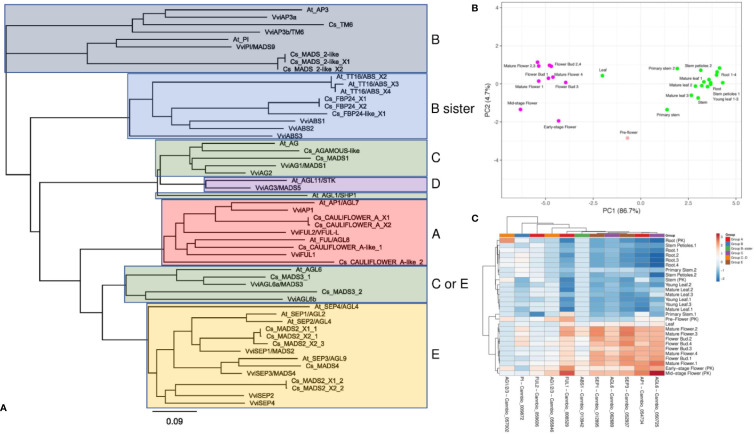
**(A)** Similarity-based neighbor-joining analysis performed using 21 amino acid sequences from the *C. sativa* (Cs) proteome (GCA_900626175.1) selected for their putative orthology (**Table 2** and, more specifically, **Supplementary Table 2**) with well-characterized ABCDE MADS box proteins belonging to *Arabidopsis thaliana* (At) and *Vitis vinifera* (Vvi). **(B)** Taking advantage of a recent in silico analysis of 31 RNA-seq datasets derived from different tissues of two different psychoactive strains (Finola and Purple Kush, NCBI SRA accession numbers: SRP006678 and SRP008673) of *C. sativa* (Massimino, 2017), a principal component analysis was performed using the expression values of the MADS box genes previously identified. The analysis is based on the ln(x+1) transformed (RPKM) values (reads per kilobase of transcript per million mapped reads) and showed a clear separation of samples related to reproductive organs from those related to vegetative organs. **(C)** Heat map showing the relative expression of each gene in the different tissues considered.

Although several monoecious varieties have been developed for agronomical purposes, in nature, *C. sativa* is a dioecious plant characterized by unisexual flowers confined to separate individuals (Chandra et al., 2017). The male flowers are pale green, carried on axillary branched cymose panicles. The panicle flowers are solitary or alternative and occur in clusters or three-flowered cymules. Each flower is composed of five tepals and as many stamens, and a thin pedicel. The tepals are ovate-oblong, 2–4 cm in length, yellowish or whitish-green, scattered, with tiny hairs. The stamens hang and consist of thin oblong and greenish filaments and anthers. The pollen grains are released through the terminal pores of the anthers (Chandra et al., 2017). Female flowers, which are dark green, subsessile and carried in pairs are closely aggregated at the apex of inflorescences, which are prevalently formed at the upper axes of branches. Every single flower is constituted of an ovary with a style that terminates in a pair of long, thin feathered stigmas at the apex, a membranous perianth surrounding the ovary and a bract. The perianth is transparent and can be smooth or partially frayed, and when mature, it covers approximately two-thirds of the ovary. The bracts are green and rough, with overlapping edges, which enclose the female flower (Chandra et al., 2017). In angiosperms, the determination of floral organs identity is regulated by a complex genetic network acting through a range of both synergistic and antagonistic interactions (Vasconcelos et al., 2009), which have been rationalized in the so-called “ABC model” (Coen and Meyerowitz, 1991). This model, described for the first time by Weigel and Meyerowitz (1994), correlates the expression of homeotic genes to specific flower structures corresponding to the four characteristic whorls of typical eudicots, the sepals (whorl 1), petals (whorl 2), stamens (whorl 3), and carpels (whorl 4). In particular, the differentiation of each flower whorl is the result of specific interactions of transcription factors (TFs) belonging to the MADS-box multigenic family, except for *APETALA 2* (*AP2*), which is part of the AP2/EREBP family (Irish, 2017). In the first stage, the model exclusively included the homeotic genes of A, B, and C classes but later it was extended to include also genes belonging to D and E classes (Jordan, 2006). A-class genes, when expressed alone, are responsible for the identity of the sepals (first whorl), while in combination with the B-class genes, they control the development of the second whorl (petals) (Jack, 2004). Female reproductive tissue (carpel) identity is specified by C-class genes, while stamen differentiation is the result of the combined interactions between B- and C-class genes (Coen and Meyerowitz, 1991). Finally, B-sister genes, closely related to the B-class, along with D-class genes, are specifically involved in determining ovule identity (Carmona et al., 2008; Vasconcelos et al., 2009). More recently, some genes exhibiting genetic redundancy and overlapping functionality (E-class) were found to form complexes with A, B, C, and D TFs (Vandenbussche et al., 2003; Castillejo et al., 2005), playing a decisive role in whorl development. In the last 20 years, the ABCDE model and the molecular bases underlying floral development have been deeply investigated and reviewed in model species such as *Arabidopsis thaliana* (Robles and Pelaz, 2005), *Antirrhinum majus* (Mizzotti et al., 2014), *Petunia hybrida* (Colombo et al., 1997), and *Vitis vinifera* (Palumbo et al., 2019b). In contrast, the application of this model in *C. sativa* has never previously been evaluated. To take the first step in this direction, we started by selecting 21 amino acid sequences from the cannabis proteome (GCA_900626175.1) based on their putative orthology (BLASTp; http://blast.ncbi.nlm.nih.gov/Blast.cgi) with well-characterized ABCDE proteins belonging to Arabidopsis and grapevine (**Supplementary Table 2**). A similarity-based neighbor-joining analysis (Geneious software v7.1.5, Biomatters, Ltd., Auckland, New Zealand) was then performed using the amino acid sequences of the three species (cannabis, Arabidopsis, and grapevine; **Figure 3A**). The phylogenetic tree demonstrates that the ABCDE TFs selected from grapevine and Arabidopsis clustered together with the putative cannabis MADS-box protein orthologs. Moreover, the organization of the resulting dendrogram in six main clades was consistent with the gene classes represented by the model, reinforcing the correlation between sequence similarity and gene function. The putative ABCDE cannabis orthologs are reported in **Table 2**. Among the A-class genes, the two isoforms of Cs_CAULIFLOWER_A (XP_030481490 and XP_030481490) clustered together with VviAP1 and AtAP1/AGL7, while Cs_CAULIFLOWER_A-like_1 seemed to be the closest relative of VviFUL1 and At_FUL/AGL8. Among the B-class genes, our phylogenetic reconstruction showed that Cs_TM6 was homologous to VviAP3a and VviAP3b/VvTM6 in grapevine and At_AP3 in Arabidopsis and that Cs_MADS_2-like, Cs_MADS_2-like_X1 and Cs_MADS_2-like_X2 were all highly related to the PISTILLATA genes of *A. thaliana* and *V. vinifera* (AtPI and VviPI/VvMADS9, respectively). In our survey, Cs_FBP24-like and the two isoforms of Cs_FBP24 represent the best candidates for the B sister class due to their tight clustering with AtTT16/ABS and the three grapevine MADS-box proteins VviABS1, VviABS2, and VviABS3. The situation for classes C and D is far from clear. According to the BLASTp analysis (**Supplementary Table 2**) and the NJ dendrogram, CsAGAMOUS-like and Cs_MADS1 could represent orthologs of the C-class genes VviAG1/MADS1 and VviAG2 (in grapevine) and AtAG and AtAGL1/SHP1 (in Arabidopsis). However, the same two cannabis proteins also represent the two closest relatives of the class-D genes VviAG3/MADS5 and AtAGL11/STK (**Supplementary Table 2**), highlighting the need for further investigation. Another aspect that needs to be elucidated is the close phylogenetic relationship between a second clade of the C-class, namely, the AG6-like/MADS3 genes, and the E-class genes. In fact, the NJ dendrogram shows that AtAGL6, VviAGL6a/MADS3 and VviAGL6b along with the putative cannabis orthologs Cs_MADS3_1 and Cs_MADS3_2 grouped together with the SEPALLATA clade (E-class). Although their capability to bind to AP1, B-class, D-class, and SEP-like MADS-box proteins was proven (Hsu et al., 2003; de Folter et al., 2005), it must be noted that the function of AGL6-like/MADS3 genes in flower development has not yet been fully elucidated (Ohmori et al., 2009; Schauer et al., 2009). However, based on their phylogenetic relationship with the SEPALLATA genes and their transcriptomic profiles recently described in grapevine flower development kinetics (Palumbo et al., 2019b), we cannot exclude that these genes belong to the E-class rather than the C-class. Finally, the last branch of the NJ tree included all the clustered SEPALLATA (SEP) genes, whose redundant involvement in petal, stamen, and carpel formation led to a revision of the first ABC model (Pelaz et al., 2000). In cannabis, based on the BLASTp alignment and the NJ tree, Cs_MADS4 and five different copies and isoforms of Cs_MADS2 form a subgroup closely linked to the SEP genes of grapevine and Arabidopsis. With the aim of gaining more evidence about the role of candidate homeotic genes identified in cannabis, we took advantage of a the recent in silico analysis of 31 RNA-seq datasets derived from one hemp strain and two different psychoactive strains, Finola and Purple Kush (NCBI SRA accession numbers: SRP006678 and SRP008673), of *C. sativa* to investigate the behavior of floral identity MADS box genes identified in the Cannio-2 genome. The analyzed tissues and organs included the shoots, roots, stem, young and mature leaves, and early, mid- and mature-stage flowers (Massimino, 2017). A principal component analysis based on the ln(x+1)-transformed reads per kilobase of transcript per million mapped reads (RPKM) values of all MADS box genes identified showed a clear separation of the samples related to the reproductive organs from those related to the vegetative organs, with PC1 explaining 86% of the variation between samples (**Figure 3B**), confirming the hypothesis that MADS box genes identified through BLASTp analysis are effectively homeotic genes involved in the determination of flower identity in *C. sativa*. The heat map in **Figure 3C** shows the relative expression of each gene in the different tissues considered. Unsupervised hierarchical clustering of samples based on gene expression values revealed two clusters of samples with specific expression patterns for MADS box genes. Cluster 1 was almost exclusively composed of samples related to reproductive organs, including flower buds (stages 1–4), mature flowers (stages 1–4), and pre, early-, and mid-stage flowers from the Purple Kush genotype. Cluster 2 was composed exclusively of vegetative organs and tissues, including the roots, leaves, stems and petioles. Only one gene (Cannbio_057002) showed a different behavior from what was expected, being highly expressed in root organs. The fact that this MADS box did not clearly cluster with a specific group of homeotic genes in the phylogenetic tree (**Figure 3C**) and was not expressed in reproductive tissues allowed us to exclude a possible role in flower determination. Unfortunately, the RNA-seq data were limited to the flower buds and whole flowers at different developmental stages, making it difficult to appreciate the variation in expression among genes belonging to different homeotic classes and, thus, expressed in different whorls.

**Table 2:** Identification of ABCDE candidate genes in *C. sativa*.

Class from ABCDE model	*Cannabis sativa* (GCA_900626175.1) best hitagainst V. vinifera and A. thalianaMADS-box TFs (BLASTp)	*Vitis vinifera* (PN40024 v1 ID)	*Arabidopsis thaliana* (Araport11)	Transcripts (GIFP00000000.1)corresponding to theGCA_900626175.1 proteins	Correspondence between GIFP00000000.1 transcripts andSRP006678/SRP008673
***Class A***	Cs_CAULIFLOWER_A X1 (XP_030481490), Cs_CAULIFLOWER_A X2 (XP_030481491)	VviAP1 (VIT_01s0011g00100)	At_AP1/AGL7 (AT1G69120)	Cannbio_054734	PK21815.1
	Cs_CAULIFLOWER_A-like_1 (XP_030485608), Cs_CAULIFLOWER_A-like_2 (XP_030485101)	VviFUL1 (VIT_17s0000g04990)	At_FUL/AGL8 (AT5G60910)	Cannbio_008529, Cannbio_059606	PK19698.1, PK01844.1
***Class B***	Cs_TM6 (XP_030499268)	VviAP3a (VIT_18s0001g13460), VviAP3b/VvTM6 (VIT_04s0023g02820)	At_AP3 (AT3G54340)	Cannbio_014948	n.a.
	Cs_MADS_2-like (XP_030484132), Cs_MADS_2-like_X1 (XP_030482855), Cs_MADS_2-like_X2 (XP_030482856)	VviPI/VvMADS9 (VIT_18s0001g0176)	At_PI (AT5G20240)	Cannbio_009872	PK22420.1
***Class B-sister***	Cs_FBP24_X1 (XP_030484437), Cs_FBP24_X2 (XP_030484436), Cs_FBP24-like_X1 (XP_030490979)	VviABS1 (VIT_10s0042g00820), VviABS2 (VIT_01s0011g01560), VviABS3 VIT_02s0025g02350	At_TT16/ABS (AT5G23260)	Cannbio_013942, Cannbio_063474	PK26778.1,
***Class C***	Cs_AGAMOUS-like (XP_030480504), Cs_MADS1 (XP_030481705)	VviAG1/MADS1 (VIT_12s0142g00360), VviAG2 (VIT_10s0003g02070)	At_SHP1/AGL1 (AT3G58780), At_AG (AT4G18960)	Cannbio_055846, Cannbio_057002	PK20142.1, PK03292.1
	Cs_MADS3_1 (XP_030487367), Cs_MADS3_2 (XP_030500965)	VviAGL6a/MADS3 (VIT_15s0048g01270), VviAGL6b (VIT_16s0022g02330)	At_AGL6 (AT2G45650)	Cannbio_062689, Cannbio_050725	PK14825.1, PK13658.1
***Class D***	Cs_AGAMOUS-like (XP_030480504), Cs_MADS1 (XP_030481705)	VviAG3/VvMADS5 (VIT_18s0041g01880)	At_STK/AGL11 (AT4G09960)	Cannbio_055846, Cannbio_057002	PK20142.1, PK03292.1
***Class E***	Cs_MADS2_X1_1 (XP_030484352), Cs_MADS2_X1_2 (XP_030492901), Cs_MADS2_X2_1 (XP_030484353), Cs_MADS2_X2_2 (XP_030492902), Cs_MADS2_X2_3 (XP_030484350), Cs_MADS4 (XP_030496177)	VviSEP1/VvMADS2 (VIT_14s0083g01050), VviSEP2 (VIT_17s0000g05000), VviSEP3/VviMADS4 (VIT_01s0010g03900), VviSEP4 (VIT_01s0011g00110)	At_ SEP1/AGL2 (AT5G15800), At_ SEP2/AGL4 (AT3G02310), At_ SEP3/AGL9 (AT1G24260), At_ SEP4/AGL3 (AT2G03710)	Cannbio_012895, Cannbio_052937,	PK08909.1, PK19420.1

By means of a BLASTp alignment against the ABCDE proteins of V. vinifera and A. thaliana, the candidate ABCDE proteins of C. sativa were retrieved from the representative proteome (GCA_900626175.1). The corresponding transcripts were then searched through a tBLASTn approach, aligning the candidate ABCDE proteins against the cannabis transcriptome shotgun assembly (GIFP00000000.1). Finally, to evaluate the expression levels of the putative ABCDE proteins in different tissues of two different cannabis strains (Finola and Purple Kush, SRP006678, and SRP008673, respectively), a BLASTn approach was applied, aligning the GIFP00000000.1 transcripts to the abovementioned RNA-seq experiments (SRP006678 and SRP008673).

## 4. An Overview of Conventional Schemes and a Glimpse Into Next-Generation Methods for Breeding Novel and Real F_1_ Hybrid Cannabis Cultivars

For many years, the development of new varieties of medical cannabis was not the exclusive preserve of breeders. Home growers who have acquired high-level skills and learned essential techniques of hybridization, selection, and cultivation have easily transitioned their activities from growing to breeding cannabis lineages. In recent decades, home growers have created most of the cannabis strains that have become popular in the market worldwide. Both medical (drug-type) and hemp (fiber-type) cultivars were traditionally developed for many years using mass selection. Cannabis varieties can then be easily preserved and multiplied *via* cuttings from individual plants that exhibit desirable traits matching a specific distinct phenotype. Propagation *via* cuttings is the main way to make prized varieties available as clones to maintain unaltered genotypes. When cannabis varieties are multiplied and commercialized through seeds, open-pollinated OP synthetics and F_1_ hybrids represent the only populations that can be reproduced sexually, giving rise to offspring characterized by morphological distinctiveness and uniformity, and genetic stability across generations. Cannabis is a dioecious (and anemophilous) species, with male and female plants exhibiting stamens and pistils in separate flowers. As a consequence, outcrossing through wind-mediated cross-pollination is the only natural reproduction system of *Cannabis* spp. The genetic structure of both natural populations and experimental breeds obtained *via* mass selection can usually be composed of a combination of highly heterozygous genotypes that share a common gene pool. Selfing is also possible and can be accomplished by artificially generating monoecious plants with unisexual flowers (i.e., reversing the sex of flowers from female to male on some branches) to induce self-pollination. Attempts were made to transform the reproductive organs of cannabis using irradiation (Nigam et al., 1981a) and streptovaricin (Nigam et al., 1981b) but the results were impractical. The successful use of other strategies, such as the feminization of male plants using ethephon (Mohan Ram and Sett, 1982b) and the masculinization of female plants with silver thiosulfate (Mohan Ram and Sett, 1982a), enabled to revolutionize breeding programs in cannabis. This latter treatment, in particular, is still largely used since thiosulfate inhibits the production of ethylene, a plant hormone that promotes the formation of female flowers. On the treated branches, the newly induced male flowers can develop anthers with viable pollen, while the other untreated branches of the plant will continue to grow female flowers. The female plants whose pistils are self-pollinated and their egg cells (X) fertilized by genetically female pollen (X) will give rise to a completely female progeny (XX). This method, exploitable for the multiplication of female plants by seeds, can be commercially more convenient than the female propagation by cuttings.

Nevertheless, sexual reproduction can originate segregating populations, genetically unstable and characterized by phenotypic variability, negative features that are not shown by clones. The only way to successfully use seeds of cannabis varieties is the one based on the development of true F_1_ hybrids by crossing genetically divergent but individually uniform parental inbreds.

In addition to this strategy for selfing, the production of highly homozygous genotypes can be achieved from full-sibling crosses performed by hand between sister-brother individuals that belong to the same progeny and share the same two parental lines.

**Figure 4:**
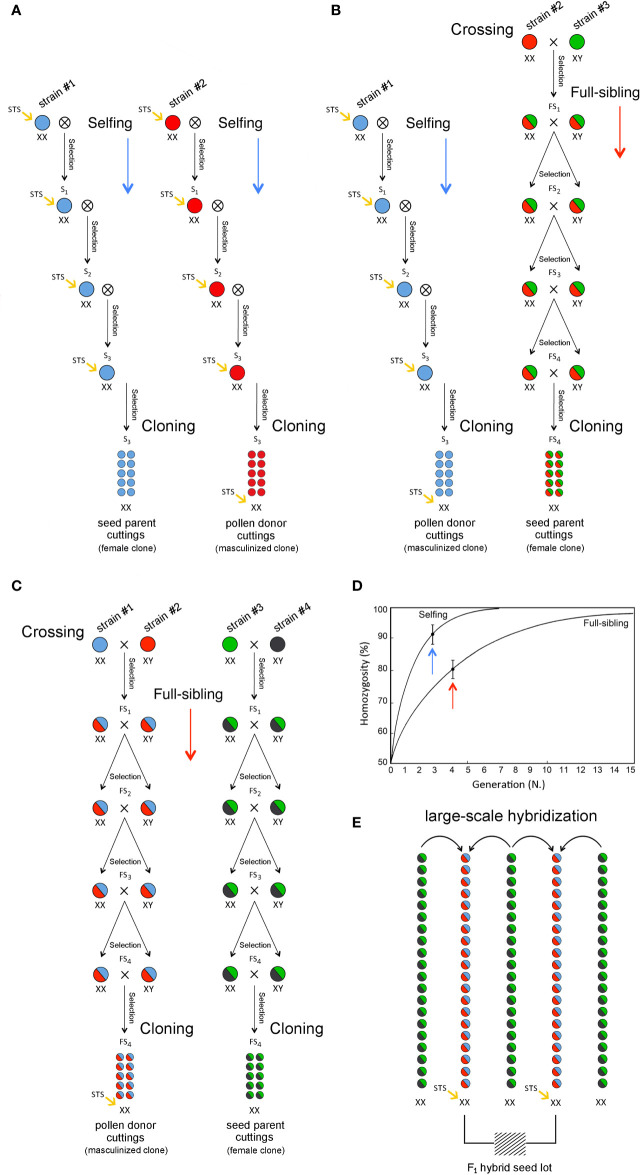
Breeding methods for the development of commercial F_1_ hybrid cultivars: two-way **(A)**, three-way **(B)** and four-way **(C)** F_1_ hybrids with inbreeding progression in case of selfing and full-sibling crosses **(D)** and large-scale hybridization and F_1_ female-seed production **(E)**.

Cannabis (sinsemilla) varieties were largely developed by crossing single male and female individuals belonging to genetically distinct landraces to create a pseudo-F_1_ hybrid. The genetic stability and uniformity of any new cultivar bred in this way can only be preserved as an individual clone through vegetative propagation through cuttings. To breed true F_1_ hybrid varieties, inbred lines stemmed *via* repeated selfing and/or full-siblings for some cycles can be used as parental stocks for the production of highly heterozygous hybrids through two-way crossing to exploit the effects of heterosis (**Figure 4**). Heterosis refers to the phenomenon in which F_1_ progeny obtained by mating two genetically divergent and antagonist inbred lines exhibit greater biomass, rate of development, and fertility than the two homozygous parents. This biological phenomenon has been extensively exploited for the development of crop varieties in several species and has been important for the development of modern fiber (hemp) cultivars but is still largely unexplored or undocumented in recreational (drug) cultivars. Since heterosis often results from the complementation in the hybrid of different deleterious (recessive) alleles that were present in one parental genotype by superior (dominant) alleles from the opposite parental genotype, the development of F_1_ hybrids usually requires progeny tests for estimating the specific combining ability (SCA) of selected inbred lines in all possible pairwise cross-combinations (diallel design). This method not only requires the selection of individual breeding parents (single female and/or male plants) but also requires that some of the progeny plants are asexually propagated *via* cuttings to perform laboratory analyses and field trials. In particular, in each generation, the selection of the most appropriate plants from either selfing or full-siblings is based on agronomic, genomic, and metabolomic investigations to choose the best individuals in terms of agronomic performance, molecular genotypes, and biochemical profiles. Selected individuals should also be used to perform parallel progeny tests aimed at determining their SCA based on F_1_ hybrid evaluation. A key step for large-scale seed production is the use of an inbred female plant (XX) as the clonal seed parent line and another genetically divergent but complementary inbred female plant (XX) that has been masculinized as the clonal pollen parent. Thus, 100% of the F_1_ hybrid seeds will be female (XX): all-female seeds are produced by cross-pollination, but all-female plants are characterized by the same highly heterozygous and vigorous genotype. The same strategy can be exploited for breeding F_1_ varieties through two-way, three-way, or four-way hybrids using two, three, or four inbred lines derived from as many parental materials/landraces (**Figure 4**) through intrasubspecific and intersubspecific hybridization. In fact, in addition to pure “indica” and “sativa” varieties, hybrid varieties with varying ratios of their genomes are common. For instance, among the most famous varieties worldwide, the “White Widow” exhibits approximately 60% “indica” and 40% “sativa” ancestry, and its plants exhibit traits from both parental biotypes. Nevertheless, the choice of the initial cross depends on the targeted cannabis market (fiber vs. drug utilization genotype and tetrahydrocannabinol/cannabidiol ratio), as some varieties are bred mostly as medicinal cannabis, and others are instead highly appreciated as recreational cannabis. Breeding for fiber production includes both monoecious and dioecious cultivars showing a high percentage of primary fibers, fast-retting phenotypes, and distinctive morphological descriptors in low-THC plants. Breeding for the production of cannabinoids comprises THC-predominant or cannabidiol (CBD)-predominant cultivars. It is worth mentioning that a limited number of cultivars have been specifically bred for seed production (Grassi and McPartland, 2017). Considering the relevance of genomics and metabolomics in the development of next-generation cannabis varieties, modern breeding methods must be based on the application of multidisciplinary skills and tools to assist professional agronomists in the evaluation or prediction, and early selection of plants with the highest potential in terms of molecular genotypes and biochemical profiles. Cannabinoids of breeding stocks can be assayed according to either quantity (i.e., percentage of cannabinoids in harvested material) or quality (i.e., THC/CBD ratio or chemotype). The quality of cannabinoids is strongly dependent on the genotype, whereas cannabinoid quantity is affected by agronomic practices, environmental conditions, and genotype x environment interactions.

## 5. Advances in Cannabis Genomics

Since the advent of genomics applied to crop plant species, breeding methods conventionally used for the development of new varieties were rearranged and readapted, as for many traits selection can be assisted by molecular markers. In particular, both simple- and multiple-locus genotyping approaches proved their utility for improving the overall genetic stability and uniformity of cultivated populations as well as for pyramiding specific genes that control resistance or tolerance to both biotic and abiotic stresses. In addition to large panels of molecular markers useful for genotyping purposes, several next-generation platforms for genome sequencing and new biotechnological techniques for gene editing are nowadays available in many crop plant species. These molecular tools allow scientists to better characterize and estimate the breeding value of plant individuals and populations using lab analyses, materials which are then used by breeders for field trials to select the superior and ideal phenotypes showing distinctiveness, uniformity, and stability.

The use of genomics in cannabis has its roots around 25 years ago with the use of dominant markers such as RFLP, RAPD, and AFLP markers (Gillan et al., 1995; Faeti et al., 1996; Jagadish et al., 1996; Forapani et al., 2001; Datwyler and Weiblen, 2006) to assess the genetic relatedness of species, varieties, and even individuals. Later on, microsatellite or SSR markers were shown to be more informative, reliable and reproducible than dominant markers for cannabis genotyping (Alghanim and Almirall, 2003; Gilmore et al., 2003; Hsieh et al., 2003). Specific marker alleles/variants were also identified as predictive and capable of discriminating hemp from marijuana (Mendoza et al., 2009). Among the most relevant microsatellite-based studies conducted on cannabis, two relatively recent researches deserve to be mentioned. In the first one, a panel of 13 SSR markers was used to test over 1,300 samples of fiber cannabis and marijuana, together with accessions from local police seizure (Dufresnes et al., 2017). In the same year, Soler et al. (Soler et al., 2017) characterized the genetic structures of 154 individuals belonging to 20 cultivars of *C. sativa* subsp. *indica* and 2 cultivars of *C. sativa* subsp. *sativa* using a set of 6 SSR markers. However, despite the number of studies conducted using dominant markers and codominant microsatellites, only Soler et al. (2017) opened to the concrete possibility of using these molecular tools for breeding goals, including the improvement and development of new varieties. Most of the studies were instead focused on germplasm management, genetic discrimination of varieties and forensic applications (e.g., drug vs. non-drug types identification).

While any marker-assisted breeding strategy in cannabis is still far to be explored, marker-assisted selection has already been successfully used. One of the main achievements that contributed the most to the shift from traditional to molecular breeding in cannabis, is the release of the first two genomes of *C. sativa* in 2011 (van Bakel et al., 2011). Since then, many studies focused on bioinformatic analyses of these genomes to mine molecular markers tightly linked to expressed genes (Gao et al., 2014) and hence useful for cannabis marker-assisted characterization and selection studies. The availability of sequenced genomes also allowed the identification and exploitation of thousands of SNP variants, which together with Genotyping-by-Sequencing (GBS) approaches, enabled the analysis of the genetic diversity of several cannabis accessions belonging to hemp and medical/recreational varieties. The use of GBS in *Cannabis* spp. has been recently described by Soorni et al. (2017), which analyzed 98 samples from two Iran germplasm collections, obtaining over 24 thousand highly informative SNPs. Also, in this case, SNP markers proved to be useful not only to classify samples belonging to different cannabis varieties but also to identify polymorphisms associated with genes belonging to the cannabinoid pathway, like *THCAS* and *CBDAS* (delta-9-tetrahydrocannabinolic acid synthase and cannabidiolic acid synthase, respectively) (van Bakel et al., 2011; Onofri et al., 2015; Weiblen et al., 2015; McKernan et al., 2020). These markers could be extremely useful in breeding programs aimed at developing new cannabis varieties for fiber production (drug-free) or medical/recreational use. Using this approach, Laverty et al. (2019) developed a physical and genetic map of *C. sativa* focusing their attention on those genes involved in the cannabinoid synthase. In particular, authors coupled the genomes of Purple Kush and Finola varieties (van Bakel et al., 2011) to the Pacific Biosciences (PacBio) long-read single-molecule real-time (SMRT) sequencing and Hi-C technology to generate a combined genetic and physical maps of cannabis. This provided new insights on the chromosome arrangement and the cannabinoid biosynthetic genes. Another milestone from the Laverty et al. (2019) study is the identification of an important gene involved in the biosynthesis of cannabichromene, a cannabinoid with a weak activity on the CB1 and CB2 receptors (involved in the neural and psychoactive effect of THC and CBD) that could be possibly used in medical therapies against pain and gastro-inflammatory diseases (Maione et al., 2011; Izzo et al., 2012; Shinjyo and Di Marzo, 2013).

More recently, based on the latest knowledge acquired on cannabis genomics, Henry et al. (2020) described the efficiency of a screening method based on KASP (Kompetitive Allele Specific PCR) technique for the identification of 22 highly informative SNPs involved in the biosynthetic pathway of cannabinoids and terpenes (important compounds for the recreational and medical cannabis industries).

It must be recognized that the increased knowledge on the most relevant cannabis biosynthetic pathways has been possible thanks to the continuous refinement of available genomes together with the public delivery of new ones. Recently, McKernan et al. (2020) sequenced and annotated 42 *Cannabis* genomes identifying SNPs useful for molecular breeding related not only to the cannabinoid synthesis but also to pathogen resistances. This could help in the production of medical/recreational cannabis without the risk of mildew contaminants that could be dangerous for consumers. In parallel, Gao et al. (2020) assembled a new genome of *C. sativa* deriving from wild samples collected in Tibet using a combination of PacBio and Hi-C technologies. Despite all these efforts, an exhaustive meta-analysis of all the cannabis genomics data published so far (Kovalchuk et al., 2020) demonstrated that the currently available cannabis genome assemblies are: i) incomplete, with approximately 10% missing, 10–25% unmapped, and centromeres and satellite sequences unrepresented; ii) ordered at a low resolution and only partially annotated for what concerns genes, partial genes, and pseudogenes. Wrapping up if, on one hand, the enormous interest raised by specific metabolic compounds (e.g., THC) has boosted the achievement of high levels of knowledge for specific biosynthetic pathways, on the other hand, the use of molecular markers for breeding new varieties is still in its embryonic phase and undoubtedly deserves further investigation to develop efficient tools transferable among laboratories. Considering the availability of a remarkable number of sequenced cannabis genomes, the starting point could be the development and implementation of an informative and representative panel of polymorphic SSR marker loci scattered throughout the genome for standardized multilocus genotyping purposes.

## 6. Characterization of Microsatellites in the Cannabis Genome and In Silico Construction of Multilocus Panels for Marker-Assisted Breeding

Cannabis genome is diploid (2*n* = 2*x* = 20) and its haploid nuclear genome size is estimated to be 818 Mbp for females (karyotype XX) and 843 Mbp for males (karyotype XY) (Sakamoto et al., 1998). The *C. sativa* plastid and mitochondrial genomes are 153,871 bp (Vergara et al., 2016b) and 415,545 bp (White et al., 2016), respectively.

Among the 12 cannabis genomes available in GenBank, 5 were assembled at the chromosome level, while the remaining ones are considered drafts at the contig (6) or scaffold (1) assembly level. The *C. sativa* cs10 genome (BioProject ID: PRJNA560384), which is the most recent, the best-assembled and, thus, considered the representative genome of this species, was chosen for microsatellites or simple sequence repeat (SSR) searches using MISA (MIcro SAtellites Identification Tool) (Thiel et al., 2003). The parameters were set as follows: minimum of 15 repetitions for mononucleotide motifs, 8 for dinucleotides, 5 for trinucleotides, and 4 for tetra-, penta-, and hexanucleotides.

A total of 126,593 perfect and 12,017 compound SSR regions were identified, with a density equal to 148 SSRs/Mbp (0.34% of the total length of the genome). This value is slightly higher but still comparable with those found for 15 other plant genomes, including *Solanum melongena*, *Capsicum annuum*, *Nicotiana tabacum*, *Petunia axillaris*, and *Coffea canephora* by Portis et al. (2018), which ranged from 60 to 140 SSRs/Mbp according to the same search parameters for SSRs (Portis et al., 2018).

Most of the SSR sequences detected in *C. sativa* exhibited a length between 15 and 19 nucleotides (60.1%), 26.5% of the sequences were 20–29 nucleotides long, 5.4% presented a length of 30–39 nucleotides and the remaining 8% were more than 40 nucleotides in length. The motif category responsible for the longest microsatellites was the dinucleotides, for which 16.7% of the sequences showed >20 repetitions and, hence, were more than 40 nucleotides long (**Supplementary Figure 1**).

**Figure 5:**
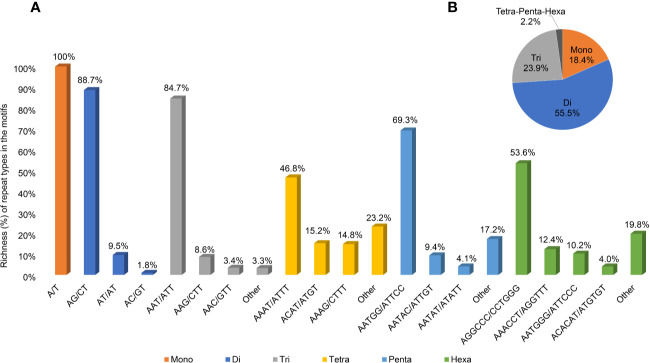
Information on SSR regions. **(A)** Abundance of the main repeat types (% base pairs among the total base pairs of the motifs) of SSR sites in the *Cannabis* cs10 genome. **(B)** Abundance of the motifs at the total SSR sites.

A second and more stringent SSR analysis was performed to identify sites suitable for genotyping analysis; longer and, putatively, more polymorphic sites were searched, increasing the stringency of the parameters to a minimum of 20 repetitions for mononucleotides, 15 for dinucleotides, 10 for trinucleotides, and 7 for tetra-, penta-, and hexanucleotides (**Supplementary Table 3**). The resulting 23,900 sequences were scored with a density of 28.2 SSRs/Mbp, with a total length equal to 0.13% of the genomic sequence. The most abundant motifs identified were the dinucleotide and the trinucleotide motifs, accounting for 55.3 and 23.9% of the total length of the SSR sequences, respectively (**Figure 5**), followed by mononucleotides motifs (18.4%), while the remaining tetra-, penta-, and hexanucleotide motifs accounted for only 2.2% of the total length (with 0.6, 0.3, and 1.3% richness, respectively).

The most abundant type of SSR repeat was A/T for mononucleotides (the only type of this motif), AG/CT for dinucleotides (88.7% of the total length of this motif category), and AAT/ATT for trinucleotides (84.4%). **Figure 5** illustrates the richness of all the main repeat types among the motifs (A) and the relative motif richness in the cannabis genome (B).

To develop a panel of SSR loci that are exploitable for marker-assisted breeding (MAB) purposes, several microsatellites were selected within each linkage group to cover the entire genome at a density equal to or greater than one SSR every 5 Mb. The selection was performed taking into consideration chromosomal position, nucleotide length, and repetitive motifs. SSR-specific primer pairs were designed using the Geneious plug-in Primer3 (Untergasser et al., 2012) following the same criteria described by Palumbo et al. (Palumbo et al., 2019a) and using the same parameters for all genomic loci to make multiplex PCR assays possible.

**Figure 6:**
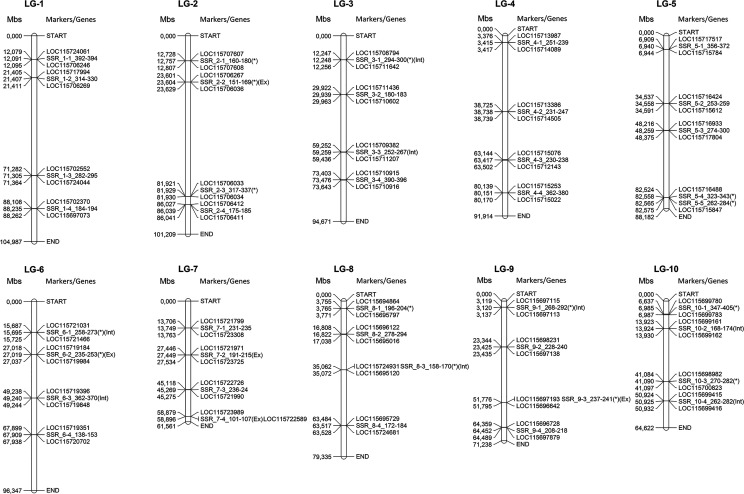
Individual linkage groups in the *Cannabis* genome (*n* = *x* = 10) with the physical position and genetic information of the selected SSR markers. Basic information on intergene and intragene sites, including intron/exon positions of SSR markers, and their corresponding physically linked genes are also reported (marker loci found to be polymorphic among all the three explored genomes are marked with an asterisk).

The panel of markers was also developed considering i) their presence in a single copy to avoid nonspecific PCR products and ii) their polymorphic nature through an in silico comparison of cs10 with two additional genomes (Finola SAMN02981385 and Purple Kush SAMN09375800). A total of 41 SSR primer pairs were designed, with an average of four per chromosome (**Figure 6** and **Supplementary Table 4** for details on chromosome accessions). Further detailed information about the selected loci is reported in **Supplementary Table 5**.

## 7. General Perspectives and Conclusions

The topic of cannabis has always aroused controversy in debates within different areas, from the ideological and political one to those more scientific of pharmacology and applied therapeutics, and even in the botanical taxonomy (Russo, 2019). Regarding the taxonomic dispute about the speciation of cannabis or lack thereof, it is unlikely to be solved because all cannabis types (whether they are considered species, subspecies, or botanical varieties) are capable of undergoing cross-hybridization and producing fertile progeny. This is intensified by the increasing number of cannabis varieties sold through the black market, along with the parallel development of legal, registered, and patented materials. Therefore, considering that morphological traits such as leaflet width and plant height do not allow a clear-cut varietal classification, biochemical profiles remain, so far, the most reliable key to characterize cannabis cultivars. In other terms, it is possible to identify cannabis types as chemical varieties (Russo, 2019). Nevertheless, these characteristics are not easy to assess analytically or stably across different environments and/or cultivation systems. Conversely, molecular markers are easy to detect and are not influenced by external factors, so they can be profitably adopted and exploited for the identification and/or authentication of *Cannabis* biotypes as molecular cultivars, including multilocus genotypes or fingerprints. Additionally, the classification of *Cannabis* through approaches involving both chloroplast DNA barcoding based on the standard genes *mat*K and *rbc*L and nuclear DNA haplotyping based on the ITS1 and ITS2 regions makes the scenario as complicated as expected. As reported in this study (**Table 1**), the number of nuclear sequences attributed to the *indica* and *ruderalis* taxa is very low, and sequences for the chloroplast genes are lacking. Moreover, the nucleotide variation found for nuclear ITS regions within each subspecies was lower than that calculated between taxonomic units, probably due to the continuous hybridization/introgression this species has undergone over time. Overall, our findings support the conclusions proposed by McPartland (McPartland, 2018), for which the *Cannabis* genus should be preferably divided into botanical varieties rather than into subspecies. Additional investigations using chloroplast DNA barcodes are needed to verify whether it is possible to detect polymorphisms or haplotypes that are useful for the authentication of cannabis taxonomies for plant varieties and their derivatives.

After several years of accelerated clandestine cultivation improvements and home-developed breeding programs, modern lines and varieties now yield dried inflorescence material that displays over 30% THC acid (THCA) by dry weight (Swift et al., 2013; Lynch et al., 2017). However, tetrahydrocannabinol is not the only cannabinoid available in high concentrations. Cultivars with considerable amounts of cannabidiolic acid (CBDA) are frequently exploited in some hashish-based products (Rustichelli et al., 1996; Hanuš et al., 2016) and are currently highly demanded spasms treatments (Devinsky et al., 2014). However, CBD and THC display contrasting neurological effects (Lynch et al., 2017). Being a non-competitive CB1/CB2 receptor antagonist (Pertwee, 2008), CBD does not own any psychoactive effect, differently from THC, whose role as a partial agonist of the two abovementioned receptors is well known.

Generally, varieties producing inappreciable or low amounts of total cannabinoids are referred to as hemp-type plants (e.g., for plant fiber and seed oil productions), while the drug-type (or therapeutic) group includes cultivars capable of synthesizing considerable levels of cannabinoids. However, from a legal point of view, THC content is the only parameter used to categorize and discriminate hemp-type from drug-type varieties. As a consequence, drug-type cannabis cultivation has been prohibited—with few recent exceptions (e.g., Canada)—since the 1930s (Lynch et al., 2017), while hemp-type lines have been continuously grown and improved in Europe and China, regardless of CBD levels. Although several investigations have shown that hemp-type cultivars are genetically distinguishable from drug-type ones (van Bakel et al., 2011), exceptions are not rare. For example, it has been observed that some hemp landraces locally cultivated in Southeast Asia are more related to local drug-type lines than to European hemp-type cultivars (Hillig, 2005). This finding reinforces the need to characterize and trace individual cannabis varieties by complementing metabolic profiles and genomic fingerprints.

Cultivated cannabis populations are usually constituted by local varieties (i.e., landraces) stemmed *via* mass selection, which shows high morphological and biochemical variation, and clones (e.g., newly bred strains) multiplied *via* cuttings from superior individual plants, which are therefore more phenotypically stable and uniform. In the past, impassionate persons rather than expert geneticists have often performed breeding activities in this species. Currently, with the advance of cannabis genetics and genomics, breeding programs are expected to include an increasing number of molecular marker-assisted selection steps. To constitute F_1_ hybrids, molecular markers such as SSR (or microsatellite-based markers) and genotyping-by-sequencing SNP (or NGS-derived) markers play a crucial role. In a notable number of crops, these molecular tools are routinely exploited to select traits of interest, to develop parental lines, and to assess their genetic stability, degree of observed homozygosity, and also their SCA. Moreover, molecular markers support the registration and thus the protection of newly developed varieties as well as the assessment of commercial F_1_ hybrids in terms of genetic identity and purity of seed stocks. Despite the huge potential, the use of these markers to improve and speed up breeding programs in cannabis has been so far surprisingly limited. Here, we bioinformatically scanned the representative cannabis genome “*C. sativa* cs10”, characterized all single-sequence repeats, and selected single-locus (polymorphic) microsatellites across all basic chromosomes. The obtained information was then used to design and implement a molecular assay for the multilocus genotyping of elite breeding stocks of cannabis using mapped microsatellite marker loci scattered throughout the genome (either in clusters or physically spaced across a single chromosome). We are confident that this method will have a great impact in the assessment of homozygosity and genetic stability of single inbred lines generated by selfing and/or full-sibling crosses and to measure the SCA between female and male inbred lines based on their genetic diversity (i.e., marker allele differentiation across different genomic loci).

Among flowering plants, the condition of separate sexes (i.e., dioecy) is relatively uncommon and it has been estimated that approximately 6% of angiosperms are dioecious (Vamosi et al., 2003). *Spinacia oleracea* (spinach), *Silene latifolia* (white campion) (Grant et al., 1994; Farbos et al., 1997), *Rumex acetosa* (sorrel) (Crossley and Ainsworth, 1993; Ainsworth et al., 1995), and *C. sativa* (hemp) are among the best characterized dioecious plant species. The molecular mechanisms that underlie dioecy are essentially unknown, but its extensive distribution across most of the Angiosperm families would suggest an independent and reiterated evolution from hermaphroditic ancestors (Charlesworth, 1985; Renner and Ricklefs, 1995). In most of the plants characterized by distinct male and female individuals, sex tissues arrest and unisexual flowers development represents a secondary stage usually preceded by an early hermaphroditic phase. Interestingly, in some taxa (including cannabis) this condition is fully reversible (e.g., through chemical products treatment) leading to the hypothesis that those genic regions involved in both sexes development remain potentially functional throughout the entire life cycle and can be regulated by genetic or epigenetic means (Di Stilio et al., 2005; Khadka et al., 2019). For instance, in the dioecious plant *Mercurialis annua* (annual mercury), it has been proved that class-B genes are mainly expressed in male flowers, while female flowers preferably accumulate transcripts from class-D genes. When male plants are feminized through cytokinin treatment, a downregulation of male-specific genes concomitant with the upregulation of female-specific genes is observed (Khadka et al., 2019). Furthermore, some species characterized by dioecy and belonging to the *Thalictrum* genus (meadow-rue) develop flowers without aborted tissues, similar to *Cannabis* species. The examination of early flower development in this species confirmed that flowers are male or female since the beginning, suggesting a homeotic-like mechanism behind sex determination. In this regard, from a comparison between *T. dioicum* (dioecious) and *T. thalictroides* (hermaphrodite), Di Stilio et al. observed duplication events affecting some the floral MADS box genes and divergence in gene regulation that may be responsible for the loss of hermaphroditism (Di Stilio et al., 2005). In the wake of these findings, we hypothesize a critical role of floral MADS box genes and homeotic sexual dimorphism in *Cannabis*. To investigate the potential of *C. sativa* as a new model for the study of dioecy and sex determination, in this study, we identified homologs of the flower organ identity genes of the ABCDE model. The putative cannabis orthologs encoding the TFs associated with the MADS-box proteins were bioinformatically characterized and demonstrated to present phylogenetic relationships with grapevine and Arabidopsis members. More evidence about the role of these genes in the ABCDE model was provided by their expression analyses: with only one exception, all were found to be highly expressed in the reproductive organs but not expressed at all in the vegetative organs. Overall, this information paves the way to further investigate the genetic factors and molecular mechanisms that underlie dioecy in *Cannabis* spp. In conclusion, we have acquired and combined data from existing genomic repositories and generated new molecular and genetic data that may be useful for basic and applied research in cannabis. In particular, our analyses and findings will continue to facilitate the development of modern breeding programs, superior variety standards, and quality assurance tools, which are still missing in the emerging legal cannabis market.

## 8. Author Contributions

The authors equally contributed to the design of the article sections and the development of the whole article, under the coordination and supervision of the P.I. GB.

## 9. Funding

This research was funded by a specific FINA project: “Breeding new varieties of Cannabis by using conventional schemes and marker-assisted selection methods” (P.I. GB).

## Conflict of Interest

The authors declare that the research was conducted in the absence of any commercial or financial relationships that could be construed as a potential conflict of interest.
