# Supplementary material for: Potentials and Challenges of Genomics for Breeding Cannabis Cultivars
Source: Front Plant Sci. 2020 Sep 25;11:573299. doi: 10.3389/fpls.2020.573299 (PMC7546024; doi:10.3389/fpls.2020.573299)
Supplement: Supplementary file 2 [file Image_1.pdf]

Supplementary Material

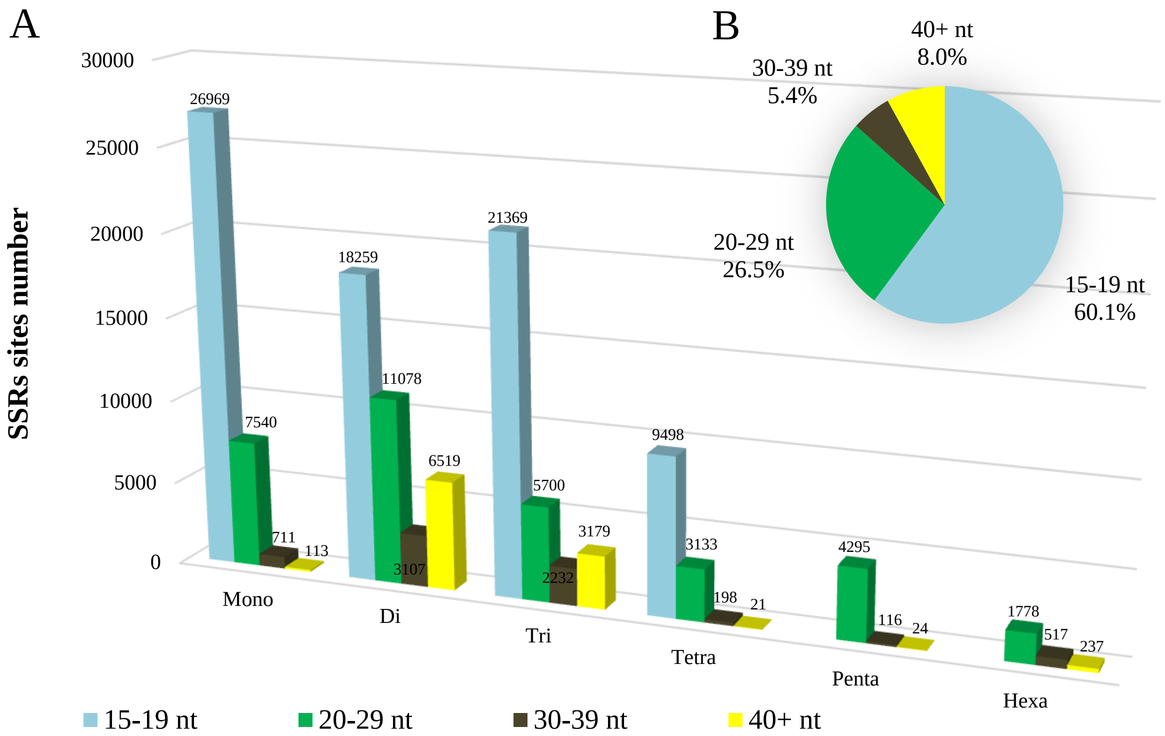

Supplementary Figure 1 (A) Number of SSRs sites of given lengths among the motifs. (B) Percentage of SSRs sites of the lengths shown (15-19, 20-29, 30-39 and >40 nucleotides) in the whole genome (*C. sativa* cs10 genome).
